# Supplementary figures and images for: An M protein coiled coil unfurls and exposes its hydrophobic core to capture LL-37
Source: eLife. 2022 Jun 21;11:e77989. doi: 10.7554/eLife.77989 (PMC9212996; doi:10.7554/eLife.77989)

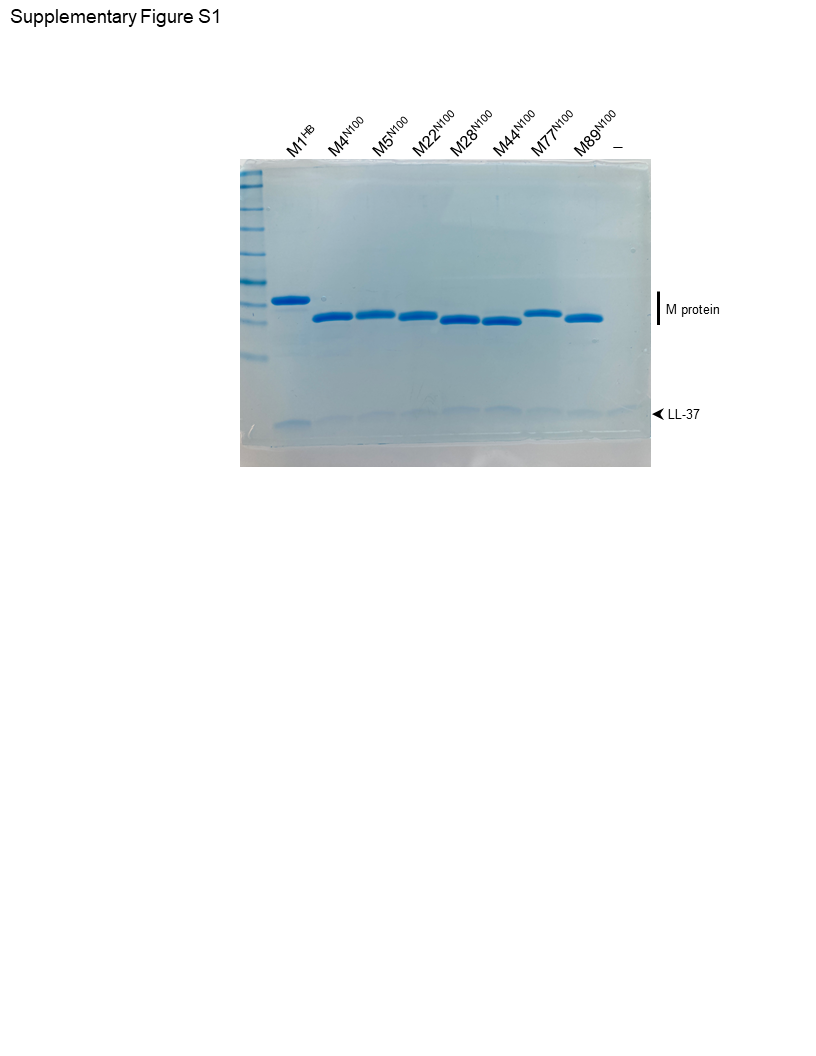

Supplement: Source data 1. [file elife-77989-data1.zip › Source Data/Figure 1 - figure supplement 1 - source data 1 labeled.tif]

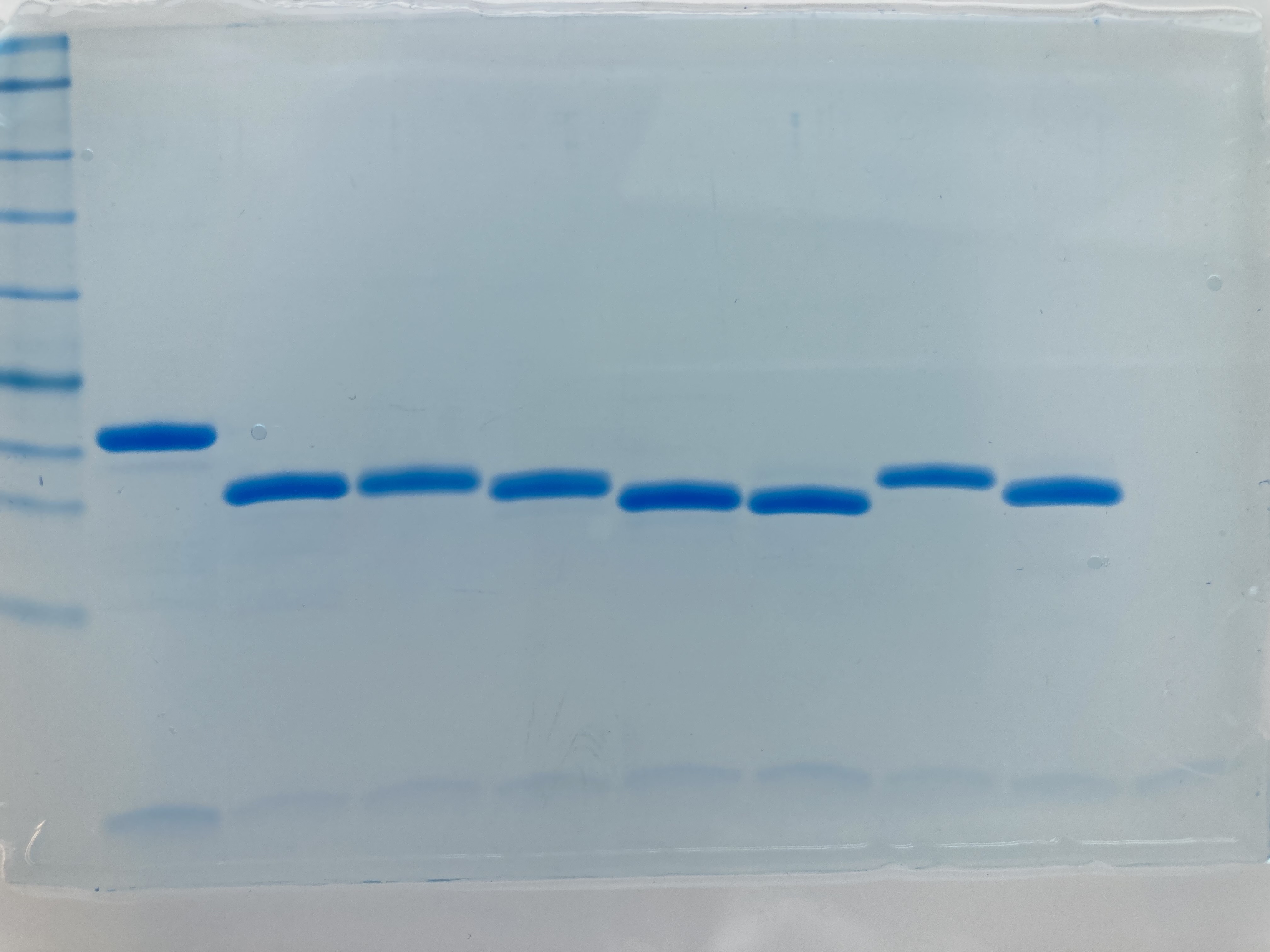

Supplement: Source data 1. [file elife-77989-data1.zip › Source Data/Figure 1 - figure supplement 1 - source data 1.jpg]

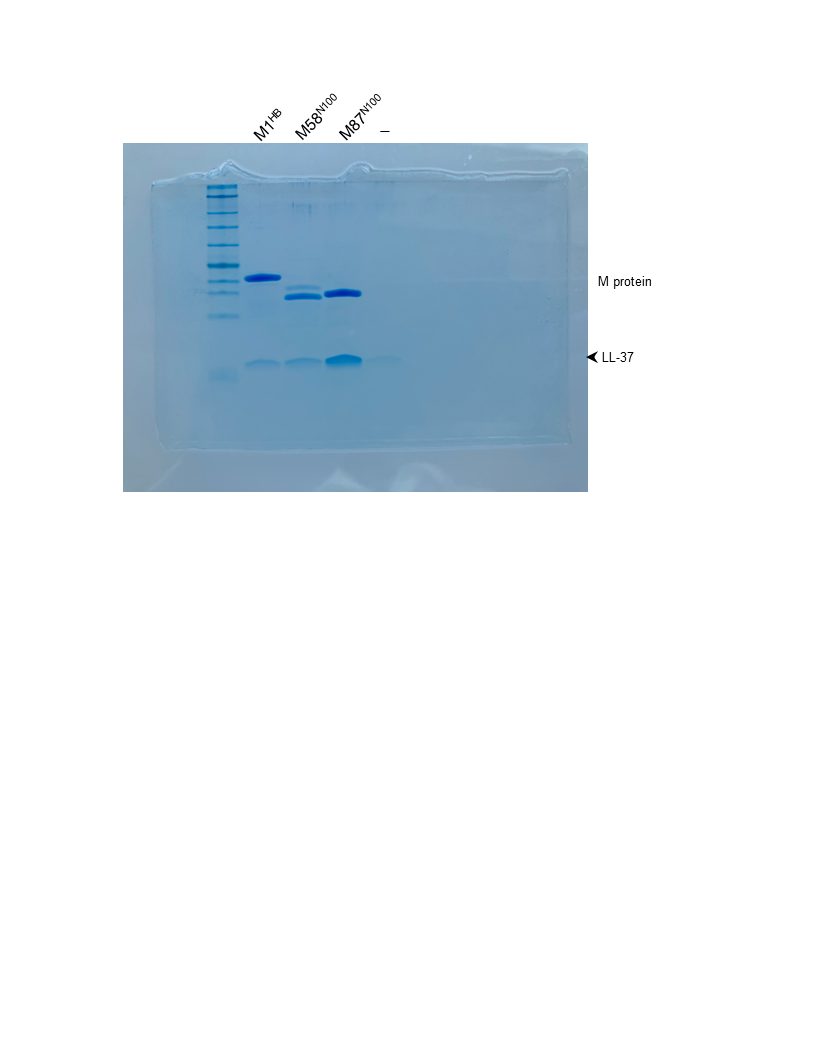

Supplement: Source data 1. [file elife-77989-data1.zip › Source Data/Figure 1 - source data 1 labeled.tif]

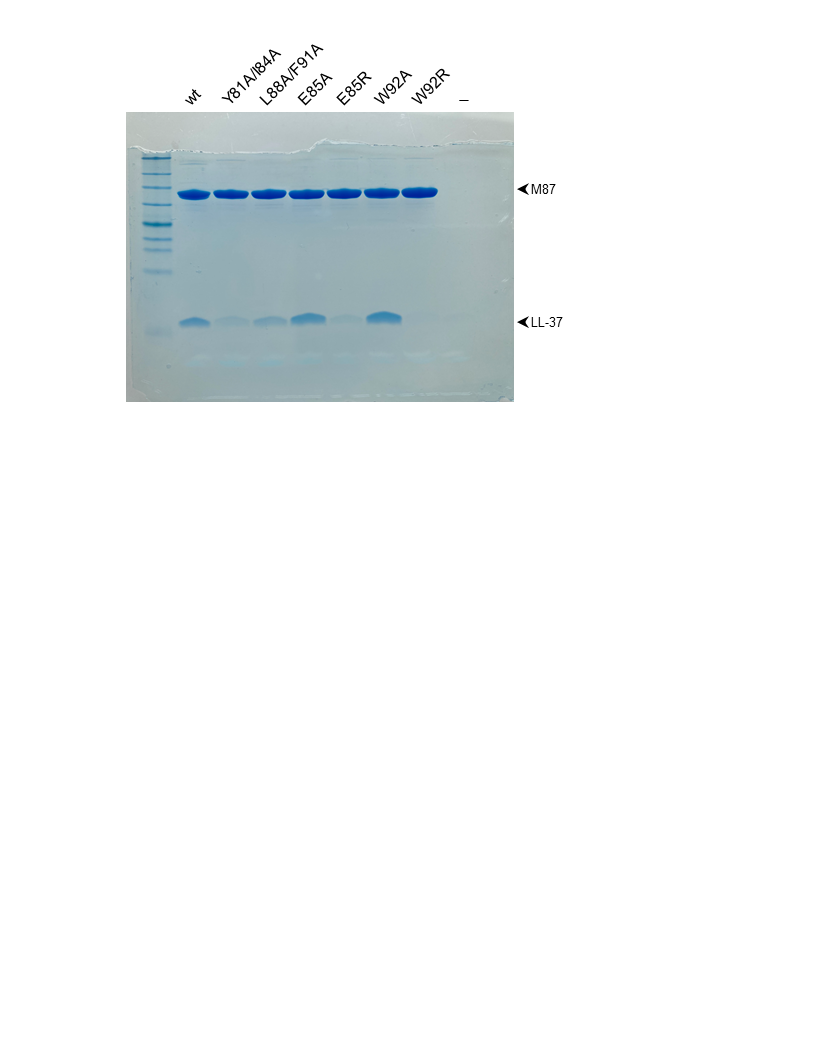

Supplement: Source data 1. [file elife-77989-data1.zip › Source Data/Figure 4 - source data 1 labeled.tif]

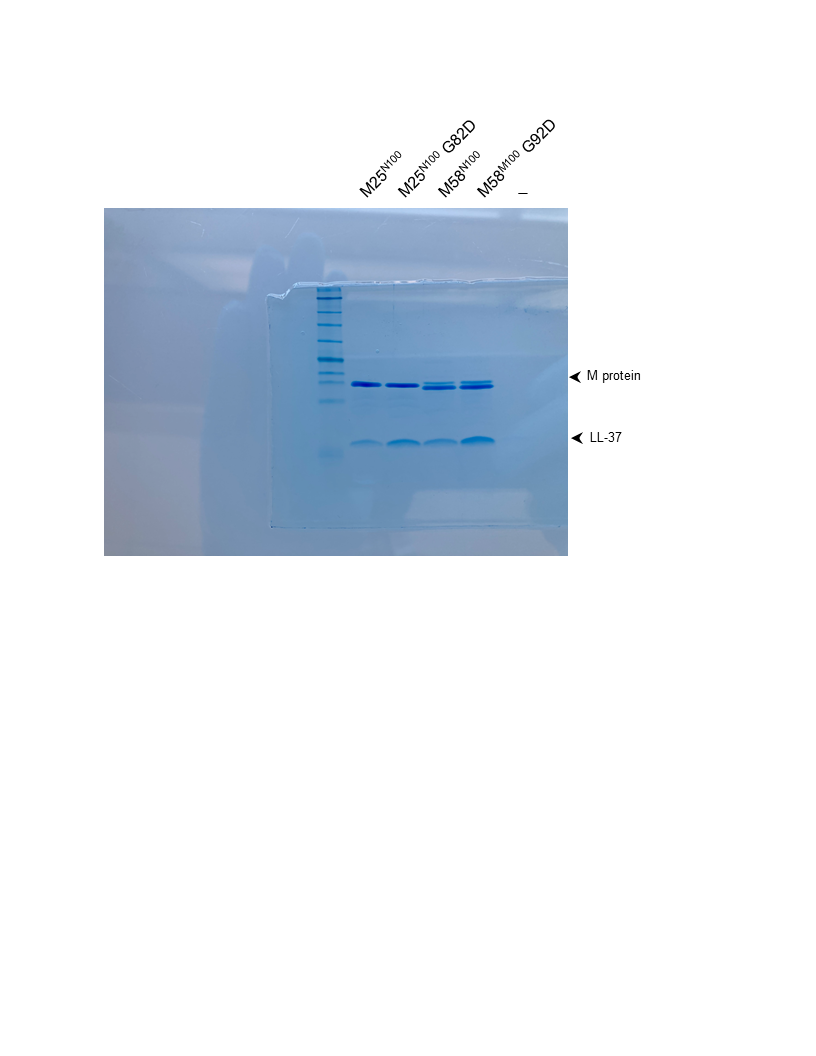

Supplement: Source data 1. [file elife-77989-data1.zip › Source Data/Figure 5 - source data 2 labeled.tif]

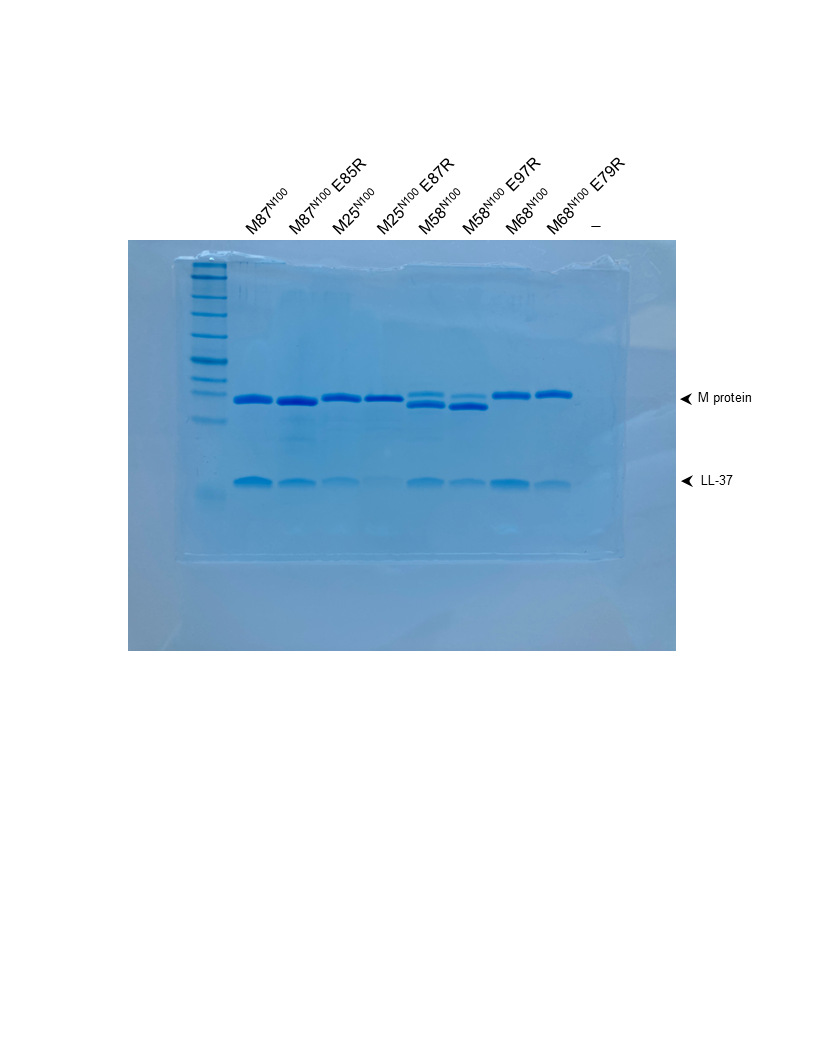

Supplement: Source data 1. [file elife-77989-data1.zip › Source Data/Figure 5 - source data 1 labeled.tif]

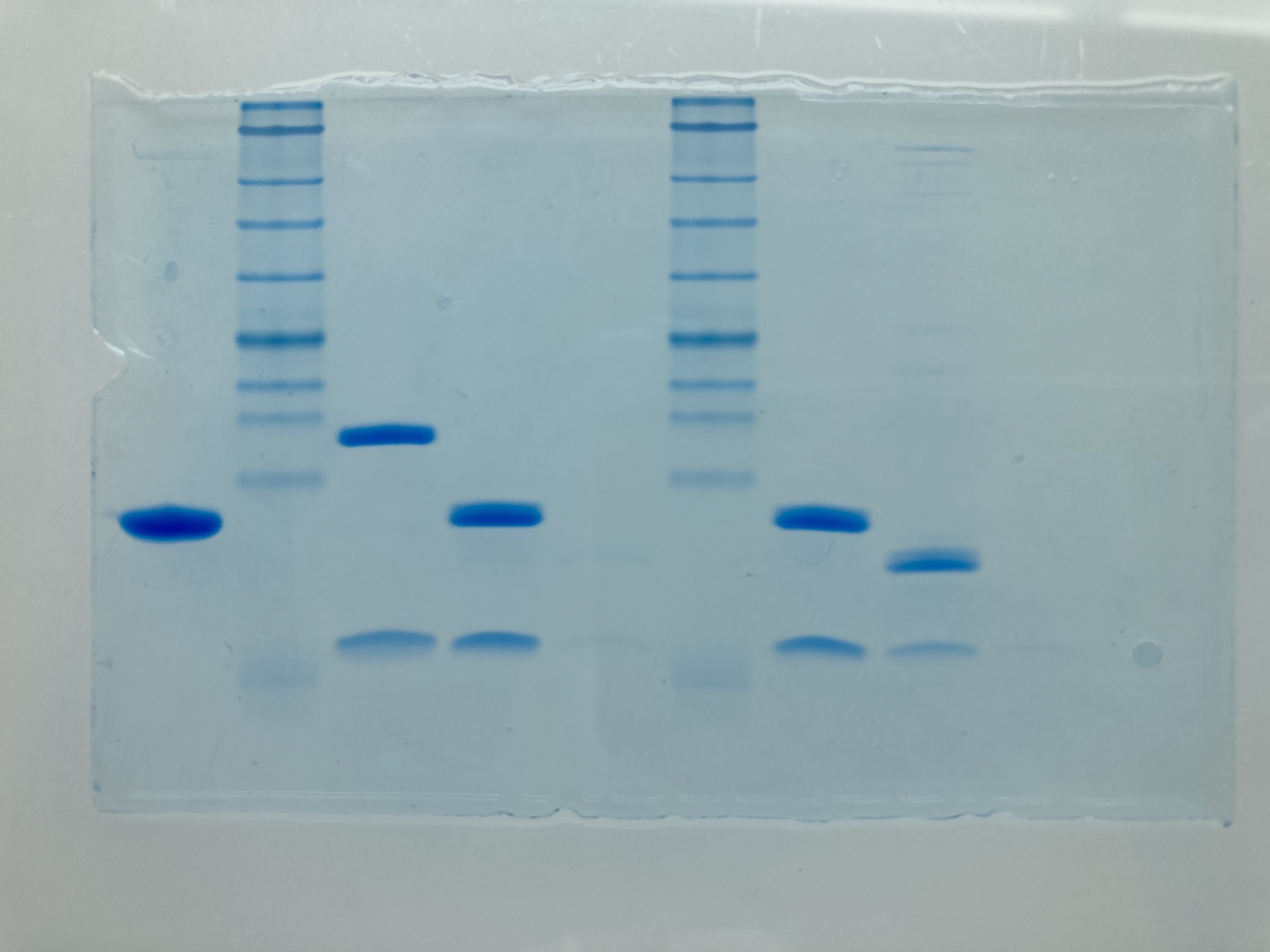

Supplement: Source data 1. [file elife-77989-data1.zip › Source Data/Figure 1 - source data 2.jpg]

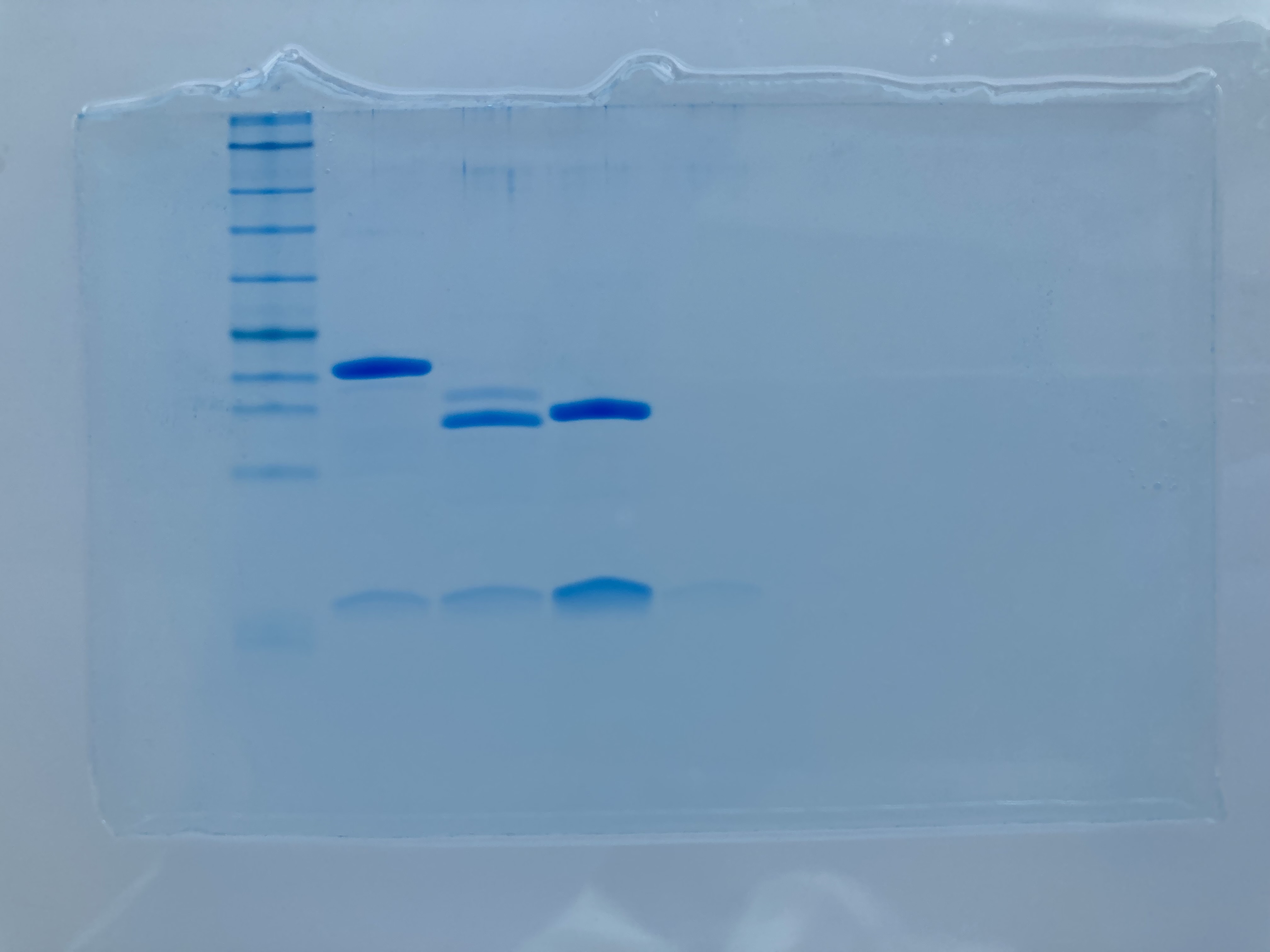

Supplement: Source data 1. [file elife-77989-data1.zip › Source Data/Figure 1 - source data 1.jpg]

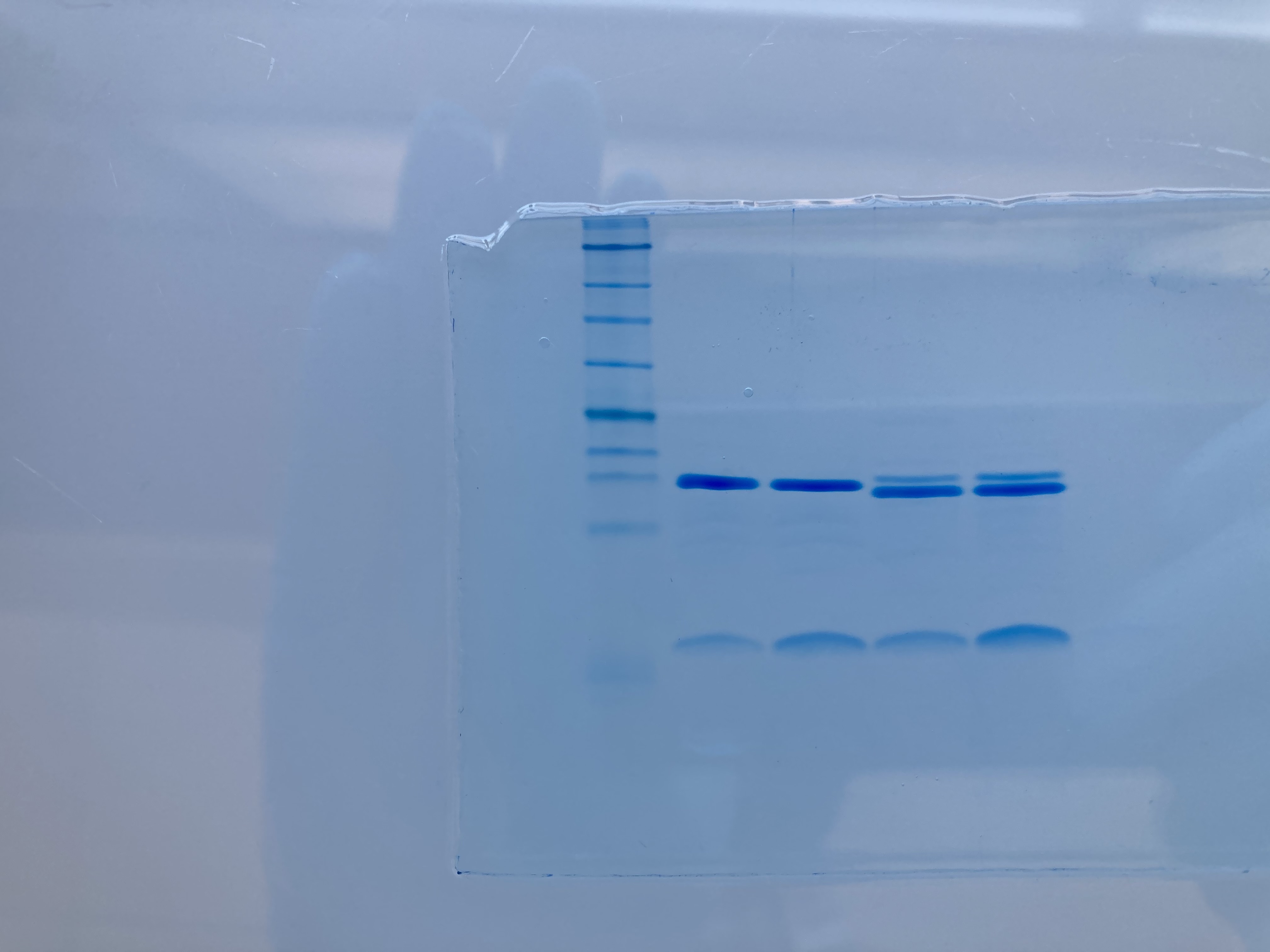

Supplement: Source data 1. [file elife-77989-data1.zip › Source Data/Figure 5 - source data 2.jpg]

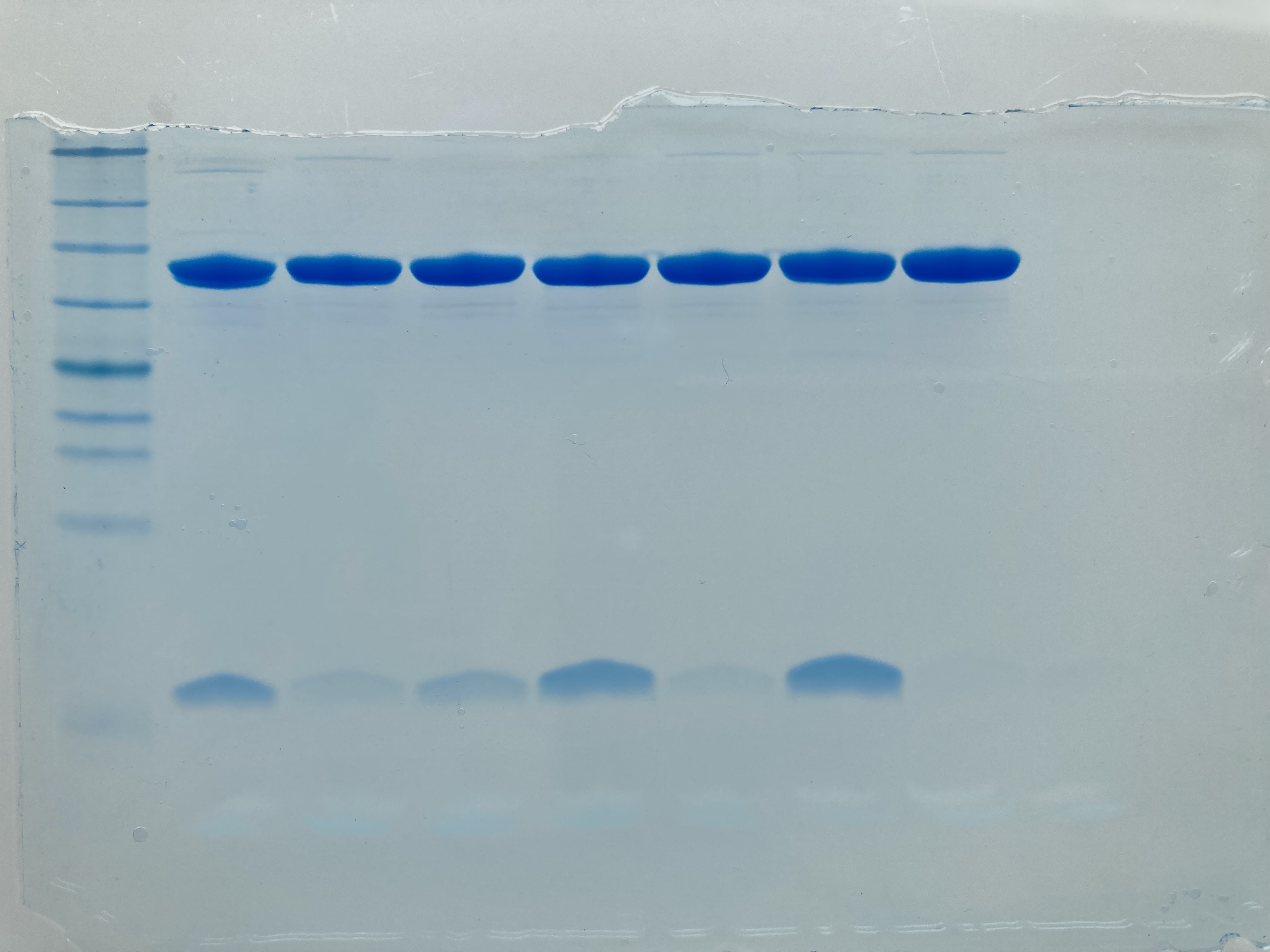

Supplement: Source data 1. [file elife-77989-data1.zip › Source Data/Figure 4 - source data 1.jpg]

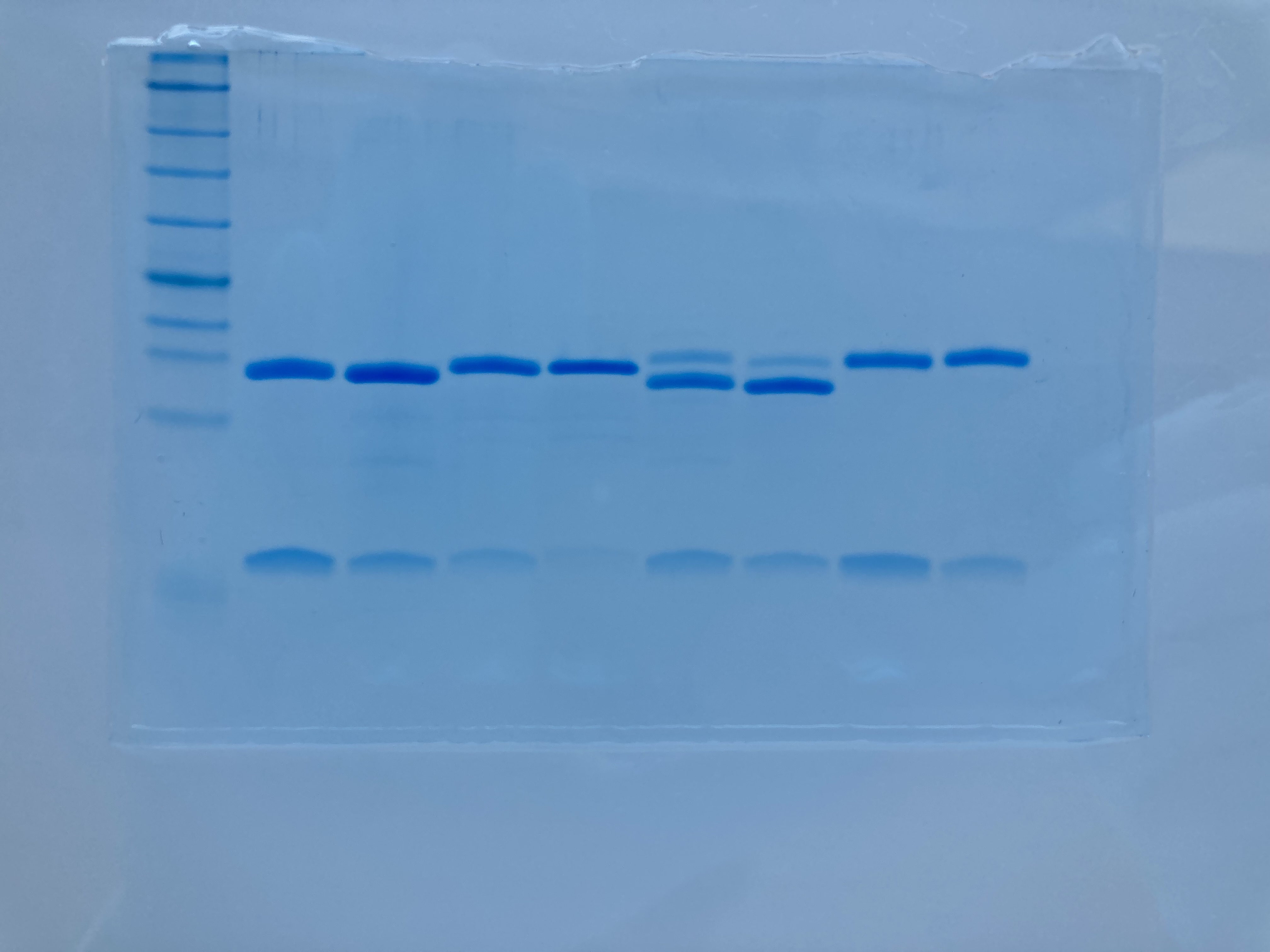

Supplement: Source data 1. [file elife-77989-data1.zip › Source Data/Figure 5 - source data 1.jpg]

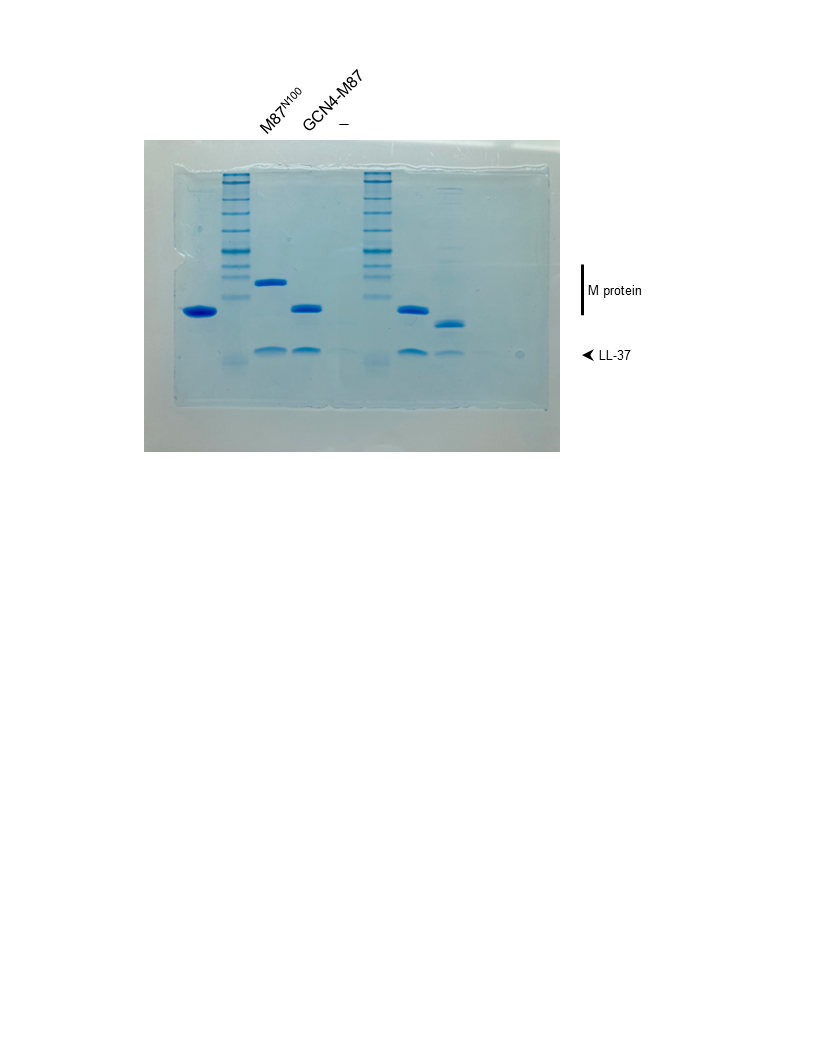

Supplement: Source data 1. [file elife-77989-data1.zip › Source Data/Figure 1 - source data 2 labeled.tif]
